# MiR-221/SIRT1/Nrf2 signal axis regulates high glucose induced apoptosis in human retinal microvascular endothelial cells

**DOI:** 10.1186/s12886-020-01559-x

**Published:** 2020-07-22

**Authors:** Bin Chen, Li Wu, Ting Cao, Hong-Mei Zheng, Tao He

**Affiliations:** Department of Ophthalmology, Renmin Hospital of Wuhan University, No. 238, Jiefang Road, Wuhan, 430060 Hubei Province P. R. China

**Keywords:** Diabetic retinopathy, miR-221, SIRT1, Nrf2, hRMEC

## Abstract

**Background:**

Diabetic retinopathy (DR) is a serious symptom associated with diabetes and could cause much suffer to patients. MiR-221, SIRT1 and Nrf2 were associated with apoptosis and proliferation and their expression were altered in DR patients. However, their roles and regulatory mechanisms in human retinal microvascular endothelial cells (hRMEC) were not clear.

**Methods:**

Expression of mRNA was detected by qRT-PCR. Protein expression was detected by Western blot. Interaction between miR-221 and SIRT1 was predicted by bioinformatics analysis and validated by dual-luciferase reporter assay. We analyzed the viability and apoptosis of hRMEC by MTT assay and FACS assay, respectively.

**Results:**

High glucose (HG) treatment enhanced expression of miR-221 and inhibited expression of SIRT1 and Nrf2. MiR-221 overexpression promoted apoptosis under HG condition. Moreover, miR-221 directly interacted with mRNA of SIRT1 and inhibited SIRT1 expression in hRMEC, through which miR-221 inhibited Nrf2 pathway and induced apoptosis of hRMEC.

**Conclusion:**

Our data demonstrated that miR-221/SIRT1/Nrf2 signal axis could promote apoptosis in hRMEC under HG conditions. This finding could provide theoretical support for future studies and may contribute to development of new treatment options to retard the process of DR development.

## Background

Diabetic retinopathy (DR) is a serious complication caused by diabetes. Serious DR would cause irreversible retina damage and may even lead to blindness. Of all patients who suffered from diabetes, around 80% would eventually develop DR. Moreover, DR related blindness would account for most blindness among population aged from 20 to 64 [1, 2]. In diabetes patients, high blood sugar, or hyperglycemia, was usually detected. Excessive blood sugar would cause damage to endothelial cells of the microvascular in retina, and this was considered the main cause of DR [3, 4]. Other hallmarks of DR, such as retinal barrier break down and capillary drop out were also related with hyperglycemia [5]. Currently, the effective therapeutic options available for DR patients were extremely limited due to lack of understanding of the regulatory mechanisms during DR development. Therefore, investigating on the regulation mechanism of human retinal microvascular endothelial cells (hRMEC) during DR development under high glucose (HG) condition bears great importance.

MicroRNAs (miRNAs) were defined as small non-coding RNA molecules with the length of 22 to 24 nt. MiRNAs could play roles in transcriptional regulation and regulate gene expression by targeting mRNAs. MiRNAs could interact with the 3’UTR (untranslated region) of mRNA and form complex with RISC (RNA induced silencing complex), and finally lead to mRNA degradation [6]. An increasing number of miRNAs were demonstrated to be related with DR development. For example, miR-15a was usually reduced during DR development and its reduction could upregulate VEGF and Robo4, which facilitated DR development [7]. MiR-150-5p, miR-30b-5p and miR-21-3p were associated with multiple angiogenesis related genes and HIF-1, which were all involved in DR regulation [8]. Besides, miR-184, miR-93-5p and more than 480 other miRNAs were reported to be differentially expressed in DR tissues and were considered related with DR regulation [9]. MiR-221 was reported by two independent groups to be progressively upregulated in DR patients, and was described as biomarker for occurrence and progression of DR [10, 11]. This suggested that miR-221 may exert critical functions in the development of DR. The function and regulation mechanism of miR-221 during DR pathogenesis, however, had not been described yet.

Sirtuin 1 (SIRT1) is a nicotinamide adenosine dinucleotide (NAD+) dependent deacetylase, which is closely related with translational modifications of many proteins. By removing the acetyl groups from transcriptional factors, SIRT1 could regulate the expression of kappa-light-chain-enhancer of B cells (NF-κB), vascular endothelial growth factor (VEGF), hypoxia-induced factors (HIFs), transforming growth factor β1 (TGF-β1) and many other cytokines, thus it was involved in multiple biological processes associated with DR progression such as apoptosis and proliferation [12]. It was reported that SIRT1 was down regulated in DR patients [13], yet its function in hRMEC remains unknown. It was also reported that SIRT1 could activate Nrf2 signal pathway and attenuate retinal damage in streptozocin (STZ) induced rat DR model [14]. Besides, multiple publications demonstrated that Nrf2 pathway was associated with apoptosis, inflammation and oxidative stress [15–17]. The function of Nrf2 in hRMEC were also enigma. Based on all these evidences, we hypothesize that miR-221 may participated in DR regulation by targeting SIRT1 and regulate Nrf2 signaling pathway.

In order to provide a new perspective for DR research, we tried to reveal the molecular mechanism of miR-221 during DR using hRMEC cell model. We confirmed that miR-221 was upregulated in HG induced hRMEC, and demonstrated that miR-221 could inhibit the viability and promote apoptosis of hRMEC. We also validated that SIRT1 was a directly target of miR-221, through which miR-221 inhibited Nrf2 expression. The regulatory effect of miR-221 on viability and apoptosis were mediated through SIRT1 and Nrf2. These findings would deepen our understanding of DR regulation and may support development of clinical therapeutic methods.

## Methods

### Cell culture

Human retinal microvascular endothelial cells (hRMEC) were obtained from the American Type Culture Collection (ATCC, Manassas, VA, USA). Cells were cultured in DMEM+ 10% foetal bovine serum (FBS) (Gibco, NY, USA) at 37 °C in a 5% CO_2_ atmosphere. For NG (Normal Glucose) or HG (High Glucose) conditions, 5 mM or 33 mM of D-glucose (Gibco) was added to the stimulation medium for 48 h, respectively. High glucose condition were defined according to previous publications and our pilot experiments [5, 18].

### Plasmids construction and transfection

MiR-221 mimics, miR-221 inhibitors, pcDNA3.1- SIRT1 (p-SIRT1), as well as their scrambled control (mimics NC, inhibitors NC, pcDNA3.1-NC) were synthesized by GenePharma (Shanghai, China). Transfection of plasmids was conveyed by lipofectamine 2000 (Invitrogen, Carlsbad, CA, USA) according to the manufacturer’s instruction.

### RNA extraction and quantitative real-time PCR

Total RNA was extracted by TRIzol reagent (Invitrogen, Carlsbad, CA, USA) according to the manufacturer’s instructions. For cDNA preparation, 1 μg of total RNA was subjected to reverse transcription by PrimeScript RT reagent Kit (Takara, Dalian, China). The amount of DNA and RNA were analyzed by Nanodrop (ThermoFisher, Waltham, MA, USA). qRT-PCR was performed on Applied Biosystems 7500 Real Time PCR System (ThermoFisher, Waltham, MA, USA) by 2 × SYBR Green master mix kit (ThermoFisher, Waltham, MA, USA) following manufactures’ instructions. Reaction volume was set as 10ul. Cycling mode was set as: 50 °C 2 min, 95 °C min, and 40 cycles of 95 °C 15 s, 60 °C 15 s, 72 °C 1 min. Relative expression was analyzed using 2^−ΔΔCt^ method and GAPDH served as internal control. All experiments were conveyed in triplicates.

### Western blot analysis

Protein were extracted by RIPA buffer (Sigma-Aldrich, Burlington, Massachusett, USA) and separated on SDS-PAGE. Proteins were then transferred onto a nitrocellulose membrane. Membranes were blocked by 5% non-fat milk dissolved in TBST buffer at room temperature for 1 h and incubated with primary antibodies overnight at 4 °C. Then, membranes were washed 3 times with washing buffer and incubated with secondary antibody for 1 h at room temperature. Signal was detected by enhanced chemiluminescence (ThermoFisher, Waltham, MA, USA). Intensity of proteins was analyzed by Image J software. All experiments were done in triplicates. Antibodies were purchased from Cell Signaling Technology (CST, Danvers, MA, USA).

### MTT assay

Cells were seeded with 100ul medium into a 96 well plate. After indicated treatments, supernatants were discarded and replaced by 20ul MTT (5 mg/ml) and placed in the incubator for another 4 h. Then, 150 ul DMSO was administrated to each well, and the plate was detected under 490 nm using microplate reader (BioTek, Hercules, CA, USA).

### Annexin-V/PI assay

Cells were collected, washed by PBS and resuspended by staining buffer. Adjust the final concentration to 1 × 10^7^ cells/mL. Take 100ul of the suspension, incubate with Annexin-V-FITC and PI (Sigma-Aldrich, USA) in dark. 15 mins later, collect cells and wash again with staining buffer. Then, cells were subjected to FACS analysis by flow cytometer (Becton Dickinson, San Jose, CA, USA).

### Dual luciferase reporter assay

Luciferase reporter vector system was obtained from Promega, Madison, Wisconsin, USA. Plasmids were constructed as previously described in the manuscript. Cells were transfected and collected 48 h post-transfection. Luciferase intensity was detected by Dual Luciferase Reporter Assay System (Promega, Madison, Wisconsin, USA) following manufacturer’s instructions. Cells were lysed with 100ul lysis buffer. Intensity was detected using GloMax20/20 luminometer (Promega, Madison, Wisconsin, USA).

### Statistical analysis

Data were analyzed with Prism 6.0 (GraphPad Software, USA). Student’s *t* test (two tailed) were performed between two groups and one-way analysis of variance (ANOVA) followed by Tukey post hoc test was performed for multiple comparison. *P* < 0.05 was considered significantly different. Data represented triplicates as mean ± standard deviation (SD).

## Results

### Expression of miR-221 in hRMEC was high while that of SIRT1 was low in high glucose condition

**Fig. 1:**
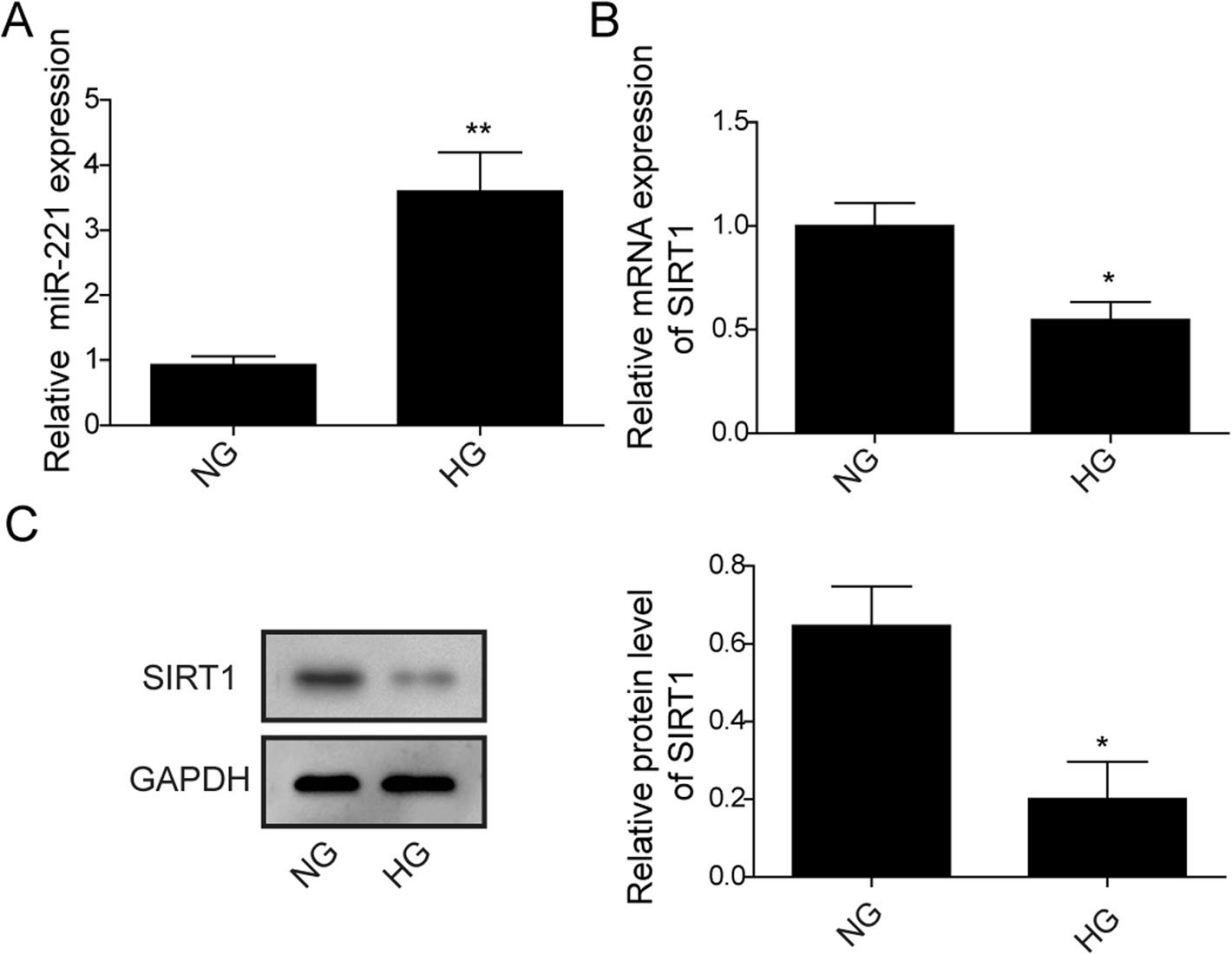
Expression of miR-221 in hRMEC was high while that of SIRT1 was low in high glucose condition. **a**: Detection of the expression of miR-221 in hRMEC under NG and HG conditions by qRT-PCR. (** indicates *P* < 0.01). **b**: Detection of SIRT1 mRNA expression in hRMEC under NG and HG conditions by RT-PCR. (* indicates *P* < 0.05). **c**: Detection of SIRT1 protein expression in hRMEC under NG and HG conditions by WB assay. Full-length blots are presented in Supplementary Figure 1C. Statistical analysis of relative SIRT1 expression level was conveyed and displayed in columns (* indicates *P* < 0.05). GAPDH served as loading control

To evaluate miR-221 and SIRT1 level in hRMEC under HG treatment, hRMEC were cultured under NG or HG condition for 48 h. The mRNA level was detected by qRT-PCR analysis and protein level was detected by Western blot analysis. The miR-221 level in cells of HG group was significantly upregulated compared to NG group (Fig. 1a). Both mRNA level and protein level were dramatically suppressed by HG treatment compared with NG group (Fig. 1b, c). We thus conclude that HG could cause miR-221 upregulation and SIRT1 downregulation in hRMEC.

### MiR-221 aggravated HG-induced apoptosis in hRMEC cells

**Fig. 2:**
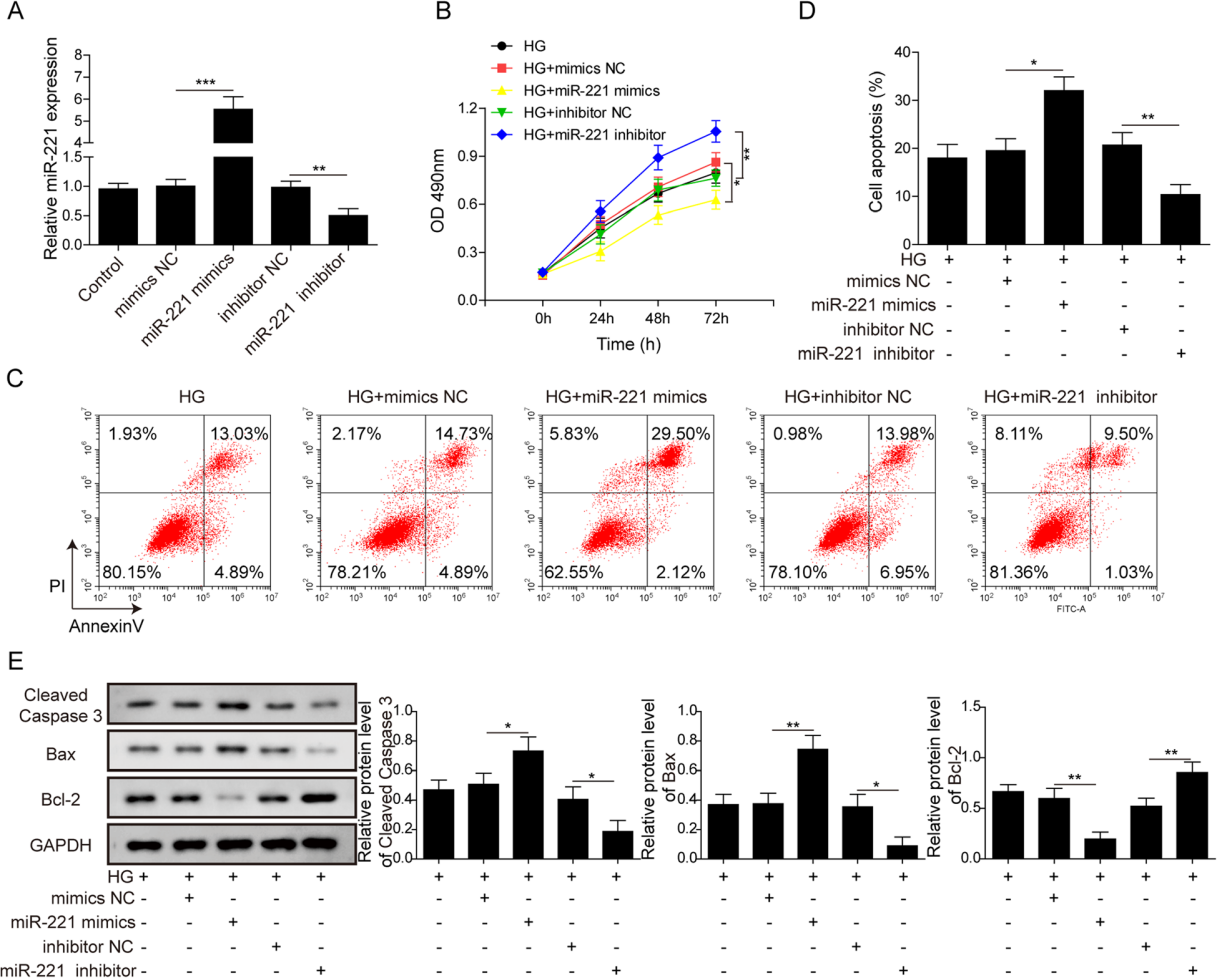
MiR-221 aggravates HG-induced apoptosis in hRMEC cells. **a**: qRT-PCR analysis of miR-221 level in hRMEC transfected with miR-221 mimics or inhibitor as indicated. (** indicates *P* < 0.01; *** indicates *P* < 0.001). **b**: MTT analysis of viability of HG included hRMEC transfected with miR-221 mimics or inhibitor as indicated. (* indicates *P* < 0.05; ** indicates P < 0.01). **c**: Apoptosis analysis of HG included hRMEC transfected with miR-221 mimics or inhibitor as indicated by FACS. **d**: Statistical analysis of FACS results in **c**. (* indicates *P* < 0.05; ** indicates *P* < 0.01). **e**: WB detection of cleaved-caspase 3, Bcl-2 and Bax expression in hRMEC transfected with miR-221 mimics or inhibitor as indicated. Full-length blots are presented in Supplementary Figure 2E. Statistical analysis of relative expression was conveyed and displayed in columns. (* indicates *P* < 0.05; ** indicates *P* < 0.01)

We next tried to evaluate whether miR-221 could affect cell viability and apoptosis of hRMEC. Mimics or inhibitor of miR-221 were successfully transfected into hRMEC and miR-221 level were detected by qRT-PCR (Fig. 2a). Cell viability was then detected by MTT assay. Over expression of miR-221 inhibited viability while miR-221 inhibitor alleviated HG induced viability inhibition (Fig. 2b). We also detected apoptosis of these cells. miR-221 overexpression significantly promoted apoptosis of hRMEC and the apoptosis rate was approximately 32% in miR-221 mimics group, while miR-221 inhibitor inhibited HG included apoptosis and the apoptosis rate was only approximately 11%. The apoptosis rate in control group, mimics NC group and inhibitor NC groups were similar to each other (approximately 18, 19 and 21%, respectively) (Fig. 2c and d). Apoptosis marker proteins were then detected by Western Blot to validate these finding. Apoptotic protein cleaved-caspase3 and Bax were significantly upregulated while anti-apoptotic protein Bcl-2 was downregulated in the miR-221 mimics group. Meanwhile, opposite results were observed in miR-221 inhibitor groups, where miR-221 inhibitor downregulated expression of cleaved caspase-3 and Bax while upregulating Bcl-2 (Fig. 2e). All these results taken together indicated that miR-221 could facilitate HG induced apoptosis in hRMEC.

### MiR-221 targeted and regulated SIRT1 expression

**Fig. 3:**
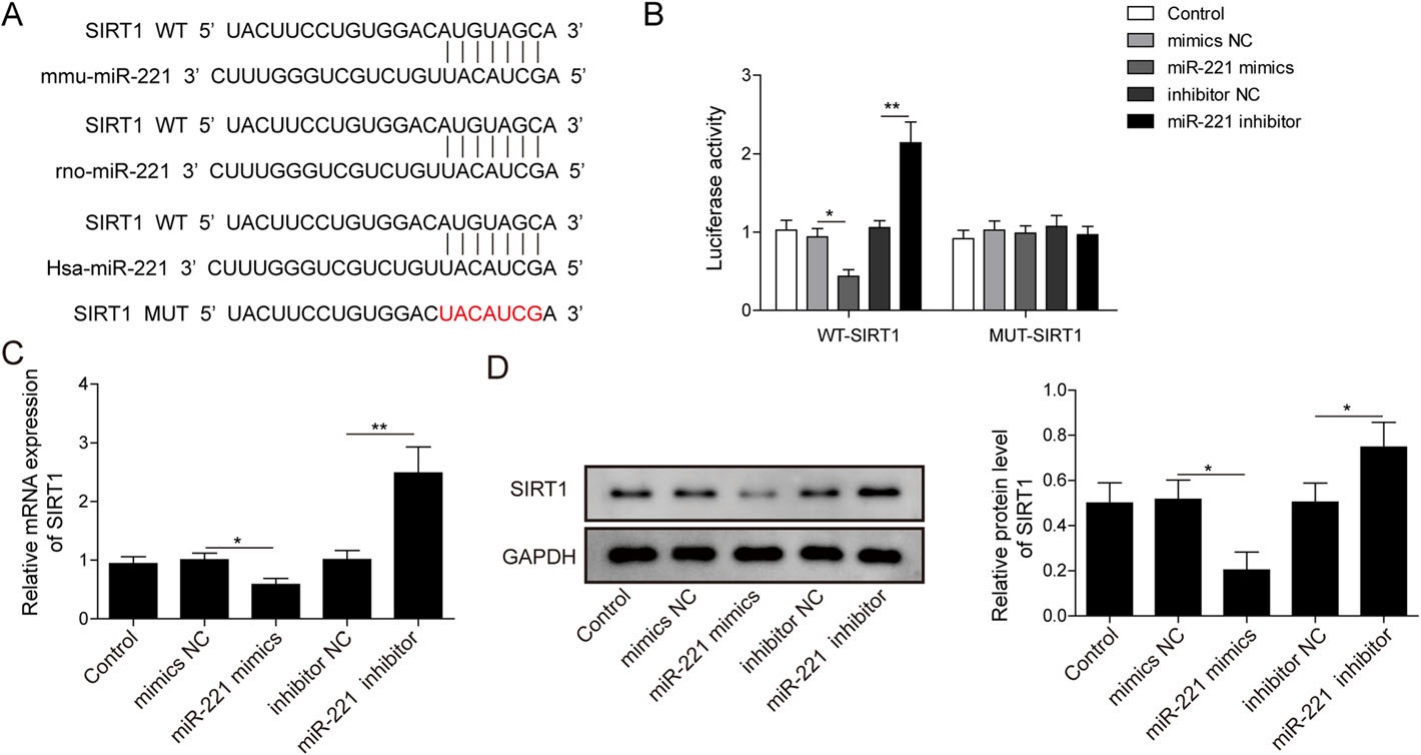
MiR-221 targeted and regulated SIRT1 expression. **a**: Bioinformatics Analysis (StarBase: http://starbase.sysu.edu.cn/) of the Binding Sites of miR-221 and SIRT1. **b**: Luciferase reporter assay in AGS cells transfected with wild type or mutated SIRT1 luciferase reporter system. (* indicates *P* < 0.05; ** indicates *P* < 0.01). **c**: qRT-PCR analysis of miR-221 level in hRMEC transfected with miR-221 inhibitor or mimics as indicated. (* indicates P < 0.05; ** indicates P < 0.01). **d**: WB detection of SIRT1 expression in hRMEC transfected with miR-221 inhibitor or mimics as indicated. Full-length blots are presented in Supplementary Figure 3D.Statistical analysis of relative expression was conveyed and displayed in columns. (* indicates *P* < 0.05)

To find out whether miR-221 directly targets SIRT1, we conveyed StarBase (http://starbase.sysu.edu.cn/)to analyze the interacting sequences between miR-221 and SIRT1. Results indicated that miR-221 could interact with the 3’untranslated region (UTR) of SIRT1 conservatively among species, such as human species, rats and mice (Fig. 3a). To validate this finding, we designed dual-luciferase reporter assay. Wild-type (WT) or mutated (MUT) 3’UTR of mRNA of SIRT1 was constructed into dual-luciferase reporter systems and the plasmid systems were transfected into hRMEC. The luciferase intensity of WT-SIRT1 was significantly decreased by miR-221 mimics and upregulated by miR-221 inhibitor, while the intensity of MUT-SIRT1 remains unaffected in neither groups (Fig. 3b). This supported the hypothesis that miR-221 could directly regulate SIRT1. We also detected mRNA and protein level of SIRT1 to support this notion. Indeed, both mRNA and protein level of SIRT1 were significantly inhibited by miR-221 overexpression, while they were both dramatically promoted by miR-221 inhibitor (Fig. 3c, d). Based on these results, we concluded that miR-221 could directly target and negatively regulate SIRT1 expression.

### MiR-221 regulated Nrf2 pathway through SIRT1

**Fig. 4:**
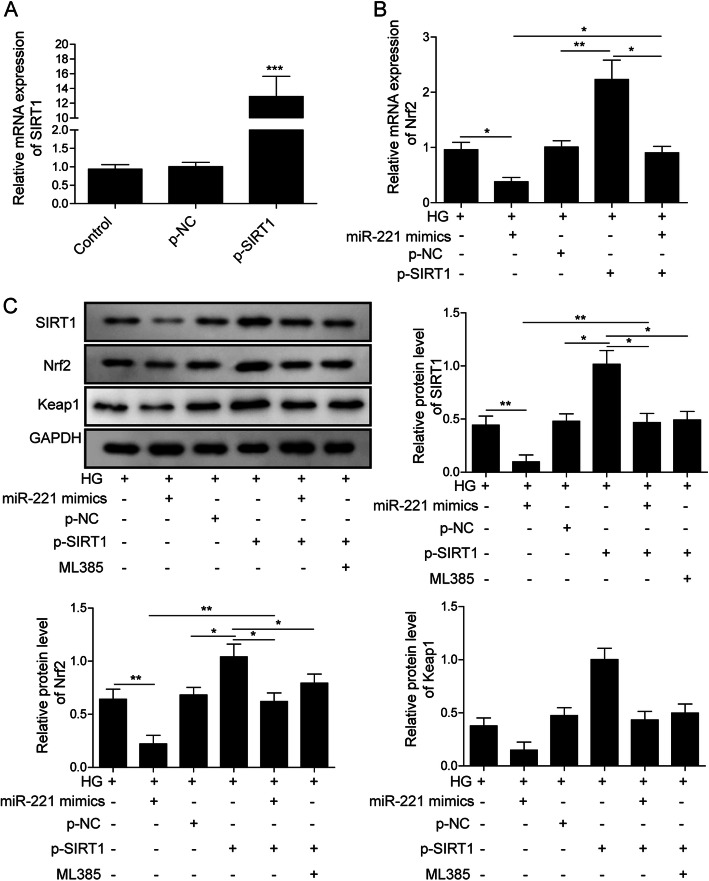
MiR-221 regulates Nrf2 pathway through SIRT1. **a**: hRMEC were transfected with pcDNA3.1-SIRT1 (p-SIRT1) as indicated. The mRNA level of SIRT1 was detected by qRT-PCR. (*** indicates P < 0.001). **b**: hRMEC were transfected with p-SIRT1 and/or miR-221 mimics as indicated and cultured under HG condition. The mRNA level of Nrf2 was detected by qRT-PCR. (* indicates P < 0.05; ** indicates *P* < 0.01). **c**: hRMEC transfected with p-SIRT1 and/or miR-221 mimics, and treated with ML385 as indicated were cultured under HG condition. Protein level of SIRT1 and Nrf2 were detected by Western Blot assay. Full-length blots are presented in Supplementary Figure 4C. Statistical analysis was conveyed and displayed in columns. (* indicates P < 0.05; ** indicates P < 0.01)

To further analyze whether miR-221 could regulate Nrf2 pathway through regulating SIRT1 expression, we transfected hRMEC with miR-221 mimics and/or pcDNA3.1-SIRT1 (p-SIRT1) under HG condition. Transfection efficiency was validated by qRT-PCR. The mRNA level of SIRT1 was significantly increased by SIRT1 transfection (Fig. 4a). Both mRNA and protein level of Nrf2 were significantly increased by SIRT1 overexpression, and additional miR-221 mimics inhibited such effect. Administration of ML385 (Nrf2 inhibitor [19]) exerted similar function with miR-221 mimics and inhibited ectopic SIRT1 induced Nrf2 upregulation. miR-221 mimics alone could inhibit both SIRT1 and Nrf2 expression (Fig. 4b, c). Besides, protein level of Keap1 was also inhibited by miR-221 mimics, which was reversed by SIRT1 overexpression (Fig. 4c). These results demonstrated that miR-221 could regulate Nrf2/Keap1 pathway through SIRT1.

### MiR-221 regulates HG induced apoptosis of hRMEC cells through SIRT1/Nrf2 pathway

**Fig. 5:**
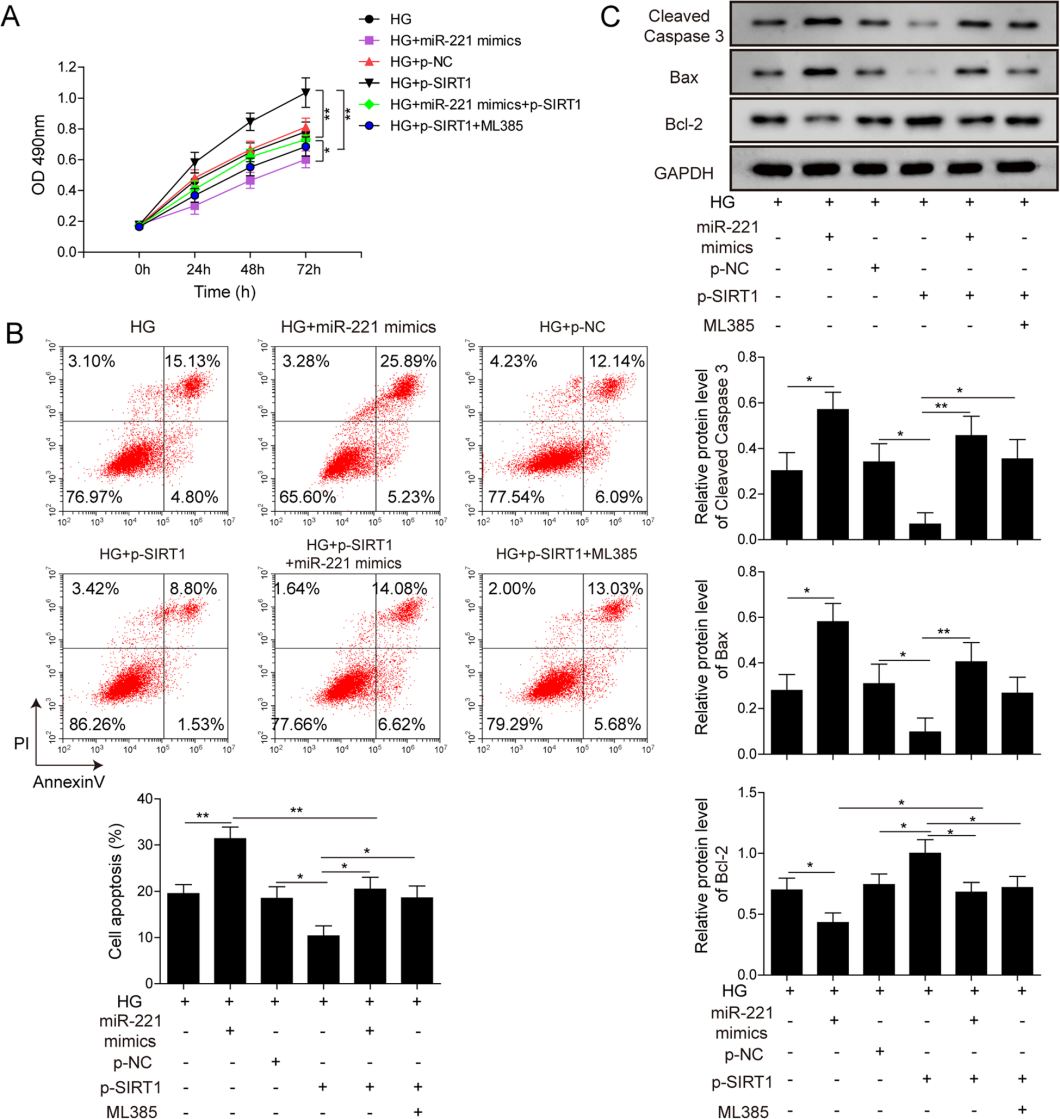
MiR-221 regulates HG induced apoptosis of hRMEC cells through SIRT1/Nrf2 pathway. **a**: hRMEC transfected with p-SIRT1 and/or miR-221 mimics, treatment with ML385 as indicated were cultured under HG condition. Viability of these cells was detected by MTT assay. (* indicates P < 0.05; ** indicates P < 0.01). **b**: hRMEC transfected with p-SIRT1 and/or miR-221 mimics, treatment with ML385 as indicated were cultured under HG condition. Apoptosis of these cells were detected by FACS assay. Statistical analysis was conveyed and displayed in columns. (* indicates P < 0.05; ** indicates P < 0.01). **c**: hRMEC transfected with p-SIRT1 and/or miR-221 mimics, treatment with ML385 as indicated were cultured under HG condition. Protein level of cleaved-caspase3, Bcl-2 and Bax were detected by Western Blot. Full-length blots are presented in Supplementary Figure 5C.Statistical analysis was conveyed and displayed in columns. (* indicates P < 0.05; ** indicates P < 0.01)

We then aimed to evaluate whether miR-221 could regulate the viability and apoptosis of hRMEC via regulating Nrf2 pathway through SIRT1. Under HG condition, hRMEC were transfected with miR-221 mimics and/or p-SIRT1. Viability of these cells was then detected by MTT assay. Results showed that overexpression of SIRT1 enhanced cell viability and counteracted the inhibitory effect of miR-221 on cell viability. Meanwhile, while SIRT1 promoted cell viability, administration of ML385 inhibited such effect of SIRT1 and restored viability to relatively normal level compared to control group (Fig. 5a). Flow cytometry results showed that overexpression of SIRT1 inhibited apoptosis and counteracted the pro-apoptotic effect of miR-221. However, the anti-apoptotic effect of SIRT1 was reversed by adding ML385 to SIRT1 overexpression group (Fig. 5b). In addition, WB analysis showed that overexpression of SIRT1 significantly decreased protein level of cleaved-caspase 3 and Bax, and up-regulated protein level of Bcl-2. What’s more, ML385 administration inhibited Nrf2 signaling pathway and reversed the regulation effects of SIRT1 on apoptosis-related proteins (Fig. 5c). These results suggest that miR-221 regulates HG-induced apoptosis in hRMEC through regulating Nrf2 pathway via SIRT1.

## Discussion

DR would happen to most diabetes patients and it was the main cause of adult blindness [20]. Currently little clinical treatment option was available to attenuate the process of DR development. Understanding the regulatory mechanism of DR and developing new treatment to retard DR progression is in urgent and unmet needs. In the present study, we investigated the expression of miR-221 in HG-treated hRMEC, studied the function of miR-221 on cell viability, apoptosis, and tried to reveal the regulatory mechanism behind these phenomena.

MiR-221 was closely associated with various biological performances, among which apoptosis and proliferation were most frequently described. For example, in pancreatic ductal adenocarcinoma, miR-221 was identified as a prognostic marker for poor outcomes [21]; in lens epithelia cells, it was reported that miR-221 could promote apoptosis [22]; in airway epithelial cell, publications also demonstrated that miR-221 could promote apoptosis and inhibit proliferation via downregulating SIRT1 [23]. All these reports indicated strong association between miR-221 and apoptosis. miR-221 was reported to be enhanced in DR patients and identified as biomarker to monitor DR development [10, 11]. However, the detailed regulatory mechanism of miR-221 during DR remains enigma. In our study, consistent with previous reports, we revealed that HG treatment upregulated expression of miR-221 in hRMEC, indicating that miR-221 held key regulatory effects during DR process.

Our further study revealed that overexpression of miR-221 promoted apoptosis and inhibited cell viability in HG induced hRMEC. Apoptotic proteins cleaved-caspase-3 and Bax were all elevated and Bcl2 was inhibited by miR-221 mimic, presumably by down regulating SIRT1. Besides hRMEC, miR-221 could regulate apoptosis and proliferation in many other cell types. For example, miR-221 could promote apoptosis of lens cells via regulating SIRT1 and E2F3 [22]. miR-221 could target suppressor of cytokine signalling-3 (SOCS3) and regulate apoptosis and proliferation via affecting JAK-STAT pathway, in bladder cancer [24]; inhibition of miR-221 alleviated LPS induced acute lung injury via inhibiting suppressor of cytokine signalling-1 (SOCS1) and apoptosis [25]; in WI-38 cells, downregulation of miR-221 resulted in relieved inflammatory damage via suppressing apoptosis [26]. However, miR-221 could also exert anti-apoptotic function in hepatocellular cancer under sorafenib treatment [27]. This might be associated with heterogeneity of genetic and epigenetic background among different cell types, especially in high mutated cancer cells. We believe that miR-221 mainly participates in DR progression regulation by regulating apoptosis and proliferation. However, more studies are still needed for validation.

Given the fact that SIRT1 was downregulated in HG induced hRMEC, and that miR-221 could regulate SIRT1 [22], we reasoned that in hRMEC miR-221 could also directly target and downregulate SIRT1. Indeed, results of bioinformatics analysis and dual luciferase reporter assay analysis all supported this notion. Besides, SIRT1 was reported to be down regulated in DR patients [13], although its regulatory mechanism was not clearly described. Since SIRT1 was reported to be associated with apoptosis and proliferation in many other cell types [12], we believe that this could be one of its roles in hRMEC and DR regulation. In Fig. 4, we validated that in hRMEC, SIRT1 could promote expression of Nrf2, which was reported to be associated with apoptosis and proliferation regulation in other cell types [15–17]. In addition, Nrf2 inhibitor ML385 could counteract with SIRT1 and reverse its effects. These all indicated that Nrf2 pathway is the main signal pathway responsible for SIRT1 induced apoptosis and proliferation regulation in hRMEC.

## Conclusions

**Fig. 6:**
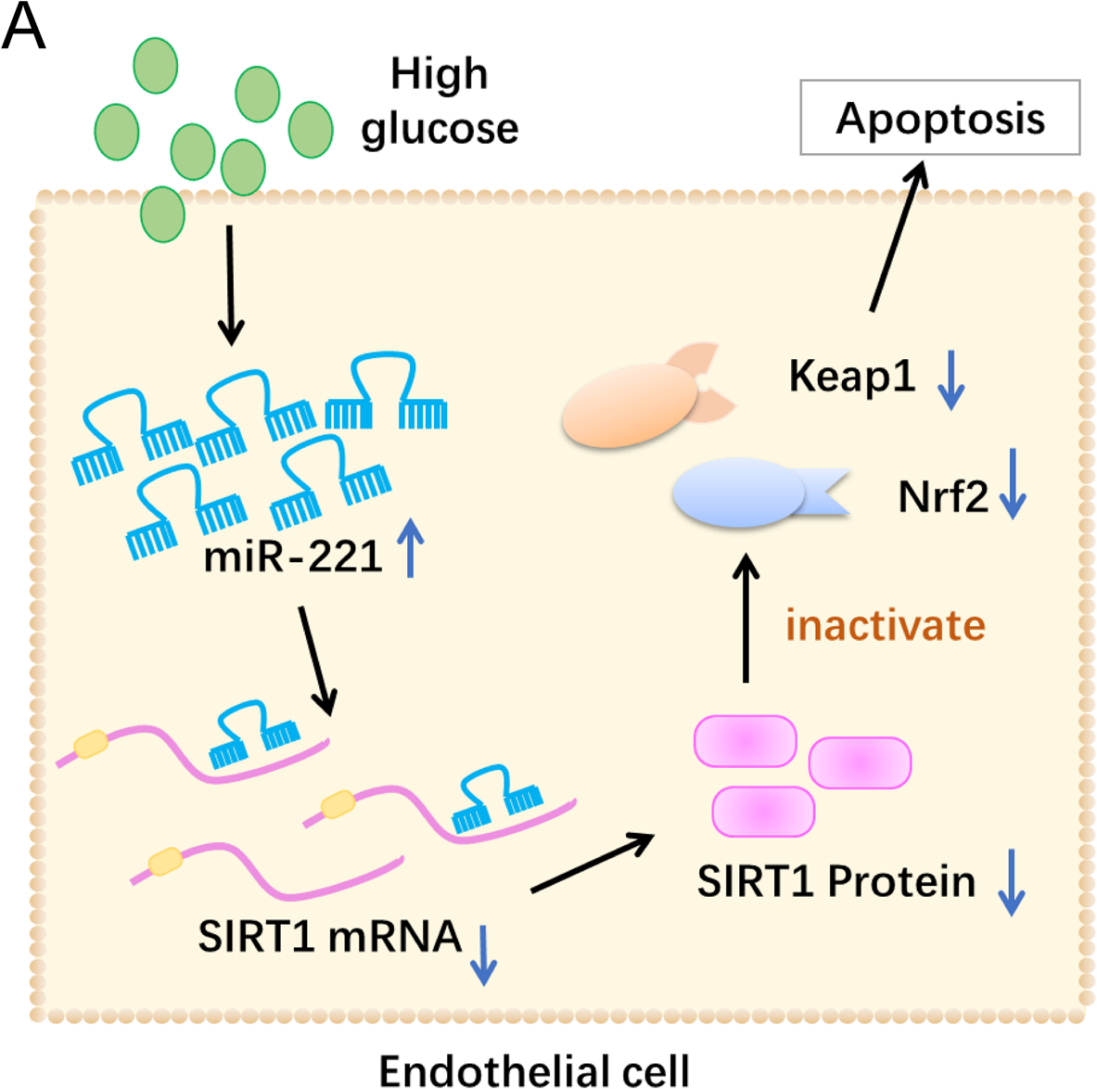
Schematic picture of miR-221, SIRT1 and Nrf2 pathway. **a**: Schematic picture was generated using Microsoft Powerpoint 2010. In endothelial cells, high glucose condition could trigger upregulation of miR-221, which could directly target and degrade mRNA of SIRT1, inhibiting its expression. SIRT1 could originally promote expression of Nrf2 and promote apoptosis. Inhibition of SIRT1 resulted in downregulated Nrf2 and Keap1, and therefore inhibited apoptosis

In this study we demonstrated that miR-221 could directly target SIRT1 and inhibit its expression, which could sequentially suppress Nrf2 signal pathway, lead to apoptosis enhancement and proliferation inhibition (Fig. 6). We believe this one of the main regulatory mechanisms of DR progression. This finding would provide a better understanding about DR progression regulation, and support future studies. Recognition of the role of miR-221/SIRT1/Nrf2 signal axis in DR regulation would also provide potential targets to develop new therapeutic methods against DR development. However, more studies are needed to achieve a more comprehensive understanding.

## Supplementary information

**Additional file 1.**

## Data Availability

The datasets used and analyzed in the present study are available from the corresponding author on reasonable request.

## Abbreviations

DR: Diabetic retinopathy
hRMEC: Human retinal microvascular endothelial cells
FACS: Flow cytometry
HG: High glucose
NG: Normal glucose
NC: Negative control
qRT-PCR: Quantitative real-time PCR
WB: Western blot
RISC: RNA induced silencing complex
UTR: untranslated region
NAD+: Nicotinamide adenosine dinucleotide
VEGF: Vascular endothelial growth factor
NF-κB: kappa-light-chain-enhancer of activated of B cells
HIF: Hypoxia - induced factors
SIRT1: Sirtuin 1
TGF-β1: Transforming growth factor β1
WT: Wild type
MUT: Mutated type
