# Supplementary figures and images for: MiR-221/SIRT1/Nrf2 signal axis regulates high glucose induced apoptosis in human retinal microvascular endothelial cells
Source: BMC Ophthalmol. 2020 Jul 22;20:300. doi: 10.1186/s12886-020-01559-x (PMC7374880; doi:10.1186/s12886-020-01559-x)

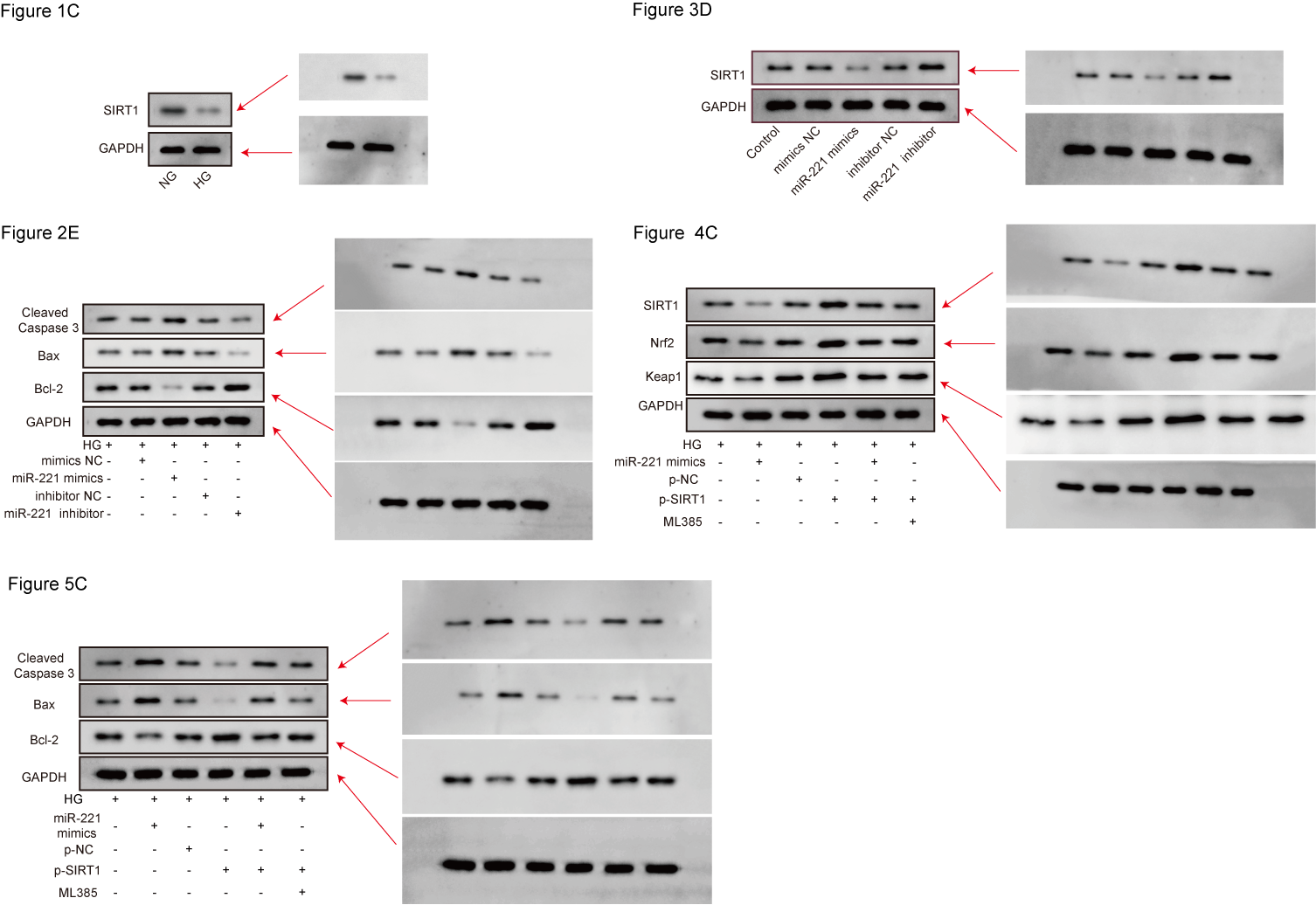

Supplement: Supplementary file 1 — Additional file 1. [file 12886_2020_1559_MOESM1_ESM.tif]
